# Effects of Neuromuscular Physiotherapy on Pain, Range of Motion, and Function in Shoulder‐Related Disorders: A Meta‐Analysis and Systematic Review

**DOI:** 10.1155/prm/3157666

**Published:** 2026-05-07

**Authors:** Yingkai Zhang, Dan Lu, Yan Lan

**Affiliations:** ^1^ Department of Orthopedics, Xianju County Hospital of Traditional Chinese Medicine, Taizhou, Zhejiang, China; ^2^ Department of Rehabilitation, TongDe Hospital of Zhejiang Province, Hangzhou, Zhejiang, China, zjtongde.com; ^3^ Department of Critical Care Medicine, Tongde Hospital of Zhejiang Province, Hangzhou, China, zjtongde.com

**Keywords:** adhesive capsulitis, proximal humerus fracture, postmastectomy lymphedema, rotator cuff tendinopathy, subacromial impingement syndrome

## Abstract

Shoulder‐related disorders are highly prevalent, with the global incidence being on the rise. Neuromuscular rehabilitation aims to optimize muscle function and motor performance by modulating the neuromuscular system, which comprises the central and peripheral nervous systems. This study aimed to synthesize evidence on neuromuscular rehabilitation for shoulder disorders, evaluating its effects on pain, range of motion, and shoulder function. English‐language literature was retrieved from the PubMed, Cochrane, and Embase databases up to June 2025 via search terms such as “Shoulder,” “Neuromuscular,” and “Rehabilitation”. The inclusion criterion was randomized controlled trials that used neuromuscular rehabilitation techniques for shoulder diseases. The primary outcome was pain improvement, and secondary indicators included joint range of motion and function. The effect size was the standardized mean difference (SMD) and 95% confidence interval (CI). Heterogeneity was evaluated via I^2^. Neuromuscular therapy significantly reduced pain (SMD [95% CI] = −2.82 [−4.79, −0.85]), with greater improvements than did the control (SMD [95% CI] = −0.75 [−1.35, −0.15]). Improvements were observed in shoulder flexion, extension, abduction, and external rotation, with external rotation showing the most significant benefit versus controls. Neuromuscular therapy improved functional scores (SMD [95% CI] = −1.95 [−3.71, −0.19]) and outperformed the control (SMD [95% CI] = −0.65 [−1.25, −0.05]). This study revealed that neuromuscular physical therapy can effectively relieve pain in patients with shoulder disorders, improve joint mobility, and enhance function. These findings support its use as an adjunctive therapy in conventional rehabilitation.

## 1. Introduction

Shoulder‐related disorders include adhesive capsulitis (frozen shoulder), subacromial impingement syndrome, rotator cuff injuries, and other disorders causing shoulder problems [1–4]. The incidence of shoulder joint diseases is increasing. The prevalence of adhesive capsulitis is 3%–5% [5]. Pain is one of the most prominent symptoms of shoulder disorders and is a major factor affecting the quality of life of patients [6, 7]. It can also lead to emotional problems such as anxiety and depression [6]. Moreover, limited shoulder joint movement is another factor affecting the quality of life of patients, as it makes it difficult for them to perform daily activities such as dressing, combing their hair, and lifting their hands [2, 3].

Currently, there are various rehabilitation treatment measures for shoulder disorders. Exercise therapy includes passive joint movements to maintain the range of motion of the joint and active muscle training to enhance muscle strength, which helps with nerve regeneration and functional recovery [3, 8–10]. Exercise therapy can not only effectively relieve pain but also improve the range of motion of patients with glenohumeral periarthritis [10]. Neuromuscular rehabilitation technology is a new type of exercise therapy [11]. In the treatment of shoulder disorders, neuromuscular rehabilitation technology has unique advantages [12]. The proprioceptive neuromuscular facilitation (PNF) technique, as a typical example of neuromuscular rehabilitation technology, stimulates the body’s proprioceptors, activates and recruits a large number of motor muscle fibers to participate in activities, adjusts the excitability of sensory nerves to change muscle tension, and alleviates muscle spasms [11, 13]. It has been applied in the treatment of various shoulder diseases.

Therefore, this study synthesizes previous research on neuromuscular rehabilitation technology in shoulder disorders to explore its effects on shoulder pain, range of motion of the shoulder joint, and function to provide more comprehensive and integrated evidence references for clinical practice.

## 2. Methods

### 2.1. Search Strategy and Study Selection

This research was prospectively registered with PROSPERO. We systematically searched the PubMed, Cochrane Central Register of Controlled Trials (by Ovid), and Embase (by Ovid) databases. The searches were restricted to articles published in the English language from the inception of each database until June 2025. We used a combination of Medical Subject Headings and free words to search, including the following terms: “Shoulder,” “Neuromuscular,” “Habilitation,” and “Rehabilitation”. OR was used to connect words with the same meaning, and AND was used to connect words with different meanings.

The inclusion criteria were determined on the basis of the PICOS framework (population, intervention, comparison, outcomes, and study design), as follows: P: shoulder‐related disorders (without limiting to specific diseases). I and C: Interventions involving the use of neuromuscular‐related physical rehabilitation techniques, with or without additional conventional rehabilitation, whereas the control group included other types of rehabilitation or other types of rehabilitation. O: The primary outcome is defined as the improvement in pain. The secondary outcomes included shoulder joint range of motion and function. S: Only randomized controlled trials (RCTs) were included.

### 2.2. Data Extraction and Risk of Bias Assessment

Two researchers independently extracted the original data and evaluated the quality of the original studies. Disagreements were resolved through discussion and consultation with an expert. The extracted data included the basic information of the original studies (authors, year, PICOS, sex, age of the population, etc.). The average values and standard deviations of the results of interest as well as the sample size were extracted. The quality of the original studies was evaluated via the revised randomized trial Cochrane risk of bias assessment tool (RoB2) [14]. If disagreements arise between the two reviewers regarding data extraction or quality assessment, they will first discuss the issues to reach a consensus. If consensus cannot be achieved through discussion, the disputed items will be submitted to a third, senior researcher in the field for independent arbitration.

### 2.3. Statistical Analysis

The statistical analysis of this study was conducted via *R* 4.5.0 software. Continuous variables are reported as the means and standard deviations. The effect size selected for this study was the standardized mean difference (SMD) and 95% confidence interval (CI). Heterogeneity was evaluated via I^2^. An appropriate pooling model was selected on the basis of the degree of heterogeneity. If heterogeneity was high (I^2^ greater than 50%), the random‐effects model was used as the main analysis to pool the effect size; otherwise, the fixed‐effects model was used as the main analysis to pool the effect size. The pooled results of another model were used for sensitivity analysis. A funnel plot was used to assess publication bias. Subgroup analysis was conducted on the basis of whether the intervention was a single neuromuscular technique (Group 1) or a combined technique with a control group (Group 2). A *p* value less than 0.05 was considered statistically significant.

## 3. Results

### 3.1. Study Characteristics

**FIGURE 1:**
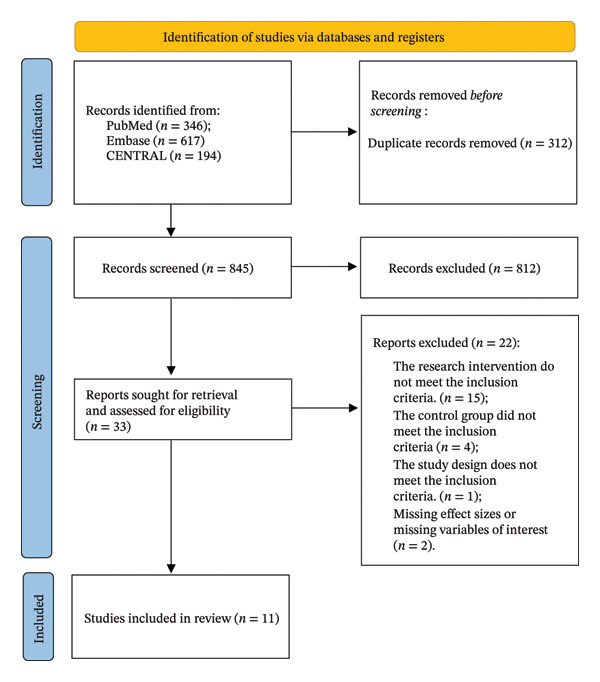
PRISMA flow diagram.

A total of 1157 records were retrieved. After duplicates were removed, initial screening and rescreening were conducted, and 11 original studies were ultimately included in the analysis [15–24]. The reasons for excluding full‐text articles are presented in the PRISMA flow diagram (Figure 1). The included studies covered multiple regions, such as China, Turkey, Canada, Bangladesh, South Korea, Brazil, and the United States. The research subjects included frozen shoulder, subacromial impingement syndrome, rotator cuff tendon disease, postmastectomy shoulder lymphedema, scapular movement disorder, proximal humeral fracture, etc., related to shoulder diseases. The intervention measures were mainly based on the PNF technique. The control group mostly adopted traditional manual therapy, strengthening training, and conventional rehabilitation. The intervention duration ranged from 1 time to 8 weeks. The study characteristics are shown in Table 1. A total of 491 patients were included, comprising 88 with frozen shoulder, 29 with subacromial impingement syndrome, 31 with rotator cuff tendinopathy, 76 with postmastectomy shoulder lymphedema, 115 with adhesive capsulitis, 56 with scapular dyskinesis, 40 with proximal humeral fractures, and 20 with general shoulder musculoskeletal disorders.

**TABLE 1:** Basic characteristics of the original studies.

Study	Year	Region	Participants	Sample size		Gender (male/female)		Age (Mean ± SD)		Intervention	Duration	Frequency	Control	Outcomes		
				*I*	*C*	*I*	*C*	*I*	*C*					Pain	Shoulder range of motion	Function/disability
Lin et al. [22]	2022	China	Frozen shoulder	24	24	10/14	11/13	52.3 ± 5.2	54.7 ± 6.5	Proprioceptive neuromuscular facilitation	4 weeks	5 times per week	Traditional manual therapy	VAS	ROM	NA
Wang et al. [16]	2023	China	Frozen shoulder	20	20	12/8	9/11	53.60 ± 5.18	54.85 ± 5.89	Neuromuscular exercises	8 weeks	5 times per week	Strengthening exercise	VAS	ROM	NA
İğrek and Çolak [23]	2022	Turkey	Subacromial impingement syndrome	15	14	6/9	2/12	47.6 ± 12.4	45.9 ± 9.7	Proprioceptive neuromuscular facilitation + conventional physiotherapy	4 weeks	5 times per week	Conventional physiotherapy	VAS	ROM	DASH
Ager ey al. [21]	2019	Canada	Rotator cuff tendinopathy	16	15	16/0	14/1	33.4 ± 9.5	36.9 ± 7.1	Neuromuscular training + usual physiotherapy care	6 weeks	3 times per week	Usual physiotherapy care	NA	NA	DASH
Guloglu et al. [18]	2023	Turkey	Mastectomy‐related lymphedema	22	22	0/22	0/22	46.0 ± 7.7	48.8 ± 9.8	Proprioceptive neuromuscular facilitation + conventional physiotherapy	8 weeks	5 times per week	Progressive resistance training	VAS	NA	DASH
Khan et al. [20]	2025	Bangladesh	Adhesive capsulitis	40	40	21/19	25/15	48 (43.25–60)	51 (45–57)	Proprioceptive neuromuscular facilitation + conventional physiotherapy	6 weeks	3 times per week	Conventional capsular stretching	SPADI	NA	SPADI
Khan et al. [20]	2021	Korea	Scapula dyskinesis	14	14	8/6	4/10	32.14 ± 6.66	32.86 ± 6.49	Proprioceptive neuromuscular facilitation	6 weeks	3 times per week	Muscle strengthening exercise	NA	NA	DASH
Khan et al. [20]	2021	Korea	Scapula dyskinesis	14	14	8/6	4/10	32.14 ± 6.66	32.01 ± 5.87	Proprioceptive neuromuscular facilitation	6 weeks	3 times per week	Muscle balance exercise	NA	NA	DASH
Balcı et al. [24]	2016	Turkey	Adhesive capsulitis	18	17	4/14	6/11	56.7 ± 7.7	58.6 ± 11.3	Proprioceptive neuromuscular facilitation + physiotherapy modalities	1 time	NA	Physiotherapy modalities	VAS	ROM	Shoulder test
Balcı et al.^∗^ [24]	2016	Turkey	Adhesive capsulitis	18	18	4/14	3/15	56.7 ± 7.7	58.1 ± 8.4	Proprioceptive neuromuscular facilitation + physiotherapy modalities	1 time	NA	Classical exercise + physiotherapy modalities	VAS	ROM	Shoulder test
da Silveira et al. [15]	2020	Brazil	Mastectomy‐related lymphedema	20	12	0/20	0/12	52.20 ± 8.314	48.417 ± 7.128	Proprioceptive neuromuscular facilitation + conventional rehabilitation	4 weeks	3 times per week	Conventional rehabilitation	NA	ROM	NA
Kus et al. [19]	2025	Turkey	Proximal humerus fracture	20	20	10/10	12/8	55.70 ± 14.30	58.35 ± 11.94	Proprioceptive neuromuscular facilitation	6 weeks	NA	Conventional therapy	VAS	ROM	DASH
Godges et al. [17]	2003	US	Shoulder musculoskeletal disorders	10	10	5/5	5/5	60.8 ± 22.3	58.6 ± 16.5	Proprioceptive neuromuscular facilitation + soft tissue mobilization	1 time	NA	Relaxing	NA	ROM	NA

*Note:* I: intervention; C: control. The specific PNF techniques applied in each study included rhythmic stabilization; dynamic and stability reversal; hold–relax; contract–relax; upper extremity diagonal movements (D1/D2); shoulder girdle patterns (flexion/extension).

Abbreviation: NA = not applicable.

^∗^Since both the intervention group and the control group combined the physiotherapy modalities, during the subgroup analysis, this group was also classified as neuromuscular rehabilitation techniques vs. control (Group 1).

### 3.2. Shoulder Pain

Six studies were included in the meta‐analysis of shoulder pain (144 participants). The results (Figure 2(a)) revealed that the severity of pain improved significantly after neuromuscular physiotherapy (SMD [95% CI] = −2.82 [−4.79, −0.85], *I* ^2^ = 96.1%). The results indicated a tendency toward improvement in shoulder pain when used alone (SMD [95% CI] = −3.16 [−6.11, −0.21], *I* ^2^ = 96.2%) or in combination with other rehabilitation methods (SMD [95% CI] = −2.50 [−5.72, 0.72], *I* ^2^ = 97.3%).

FIGURE 2Forest plots of shoulder pain outcomes. (a) Symptom improvement after neuromuscular physiotherapy. The overall pooled random‐effects SMD was −2.82 (95% CI: −4.79, −0.85), indicating a significant reduction in pain. Heterogeneity was high (*I* ^2^ = 96.1%, *τ* ^2^ = 5.8557, *p* < 0.0001). Subgroup analysis showed similar improvements when the intervention was used alone (Group 1: SMD = −3.16, 95% CI: −6.11, −0.21; *I* ^2^ = 96.2%, *τ* ^2^ = 6.5225, *p* < 0.0001) or in combination with other methods (Group 2: SMD = −2.50, 95% CI: −5.72, 0.72; *I* ^2^ = 97.3%, *I* ^2^ = 7.9364, *p* < 0.0001). The test for subgroup differences (random effects) was not significant (*χ* ^2^_1_ = 0.09, *p* = 0.7656). (b) Comparison with the control group after treatment. The overall pooled random‐effects SMD was −0.75 (95% CI: −1.35, −0.15), demonstrating that neuromuscular physiotherapy provided greater pain relief than the control. Heterogeneity was substantial (*I* ^2^ = 86.4%, *τ* ^2^ = 0.2715, *p* < 0.0001). Subgroup analysis showed a benefit for the technique alone (Group 1: SMD = −0.58, 95% CI: −0.89, −0.26; *I* ^2^ = 26.4%, *τ* ^2^ = 0.0401, *p* = 0.2532), while the analysis for the combination subgroup (Group 2: SMD = −0.92, 95% CI: −2.13, 0.28; *I* ^2^ = 93.1%, *τ* ^2^ = 0.9394, *p* < 0.0001) showed a nonsignificant trend. The test for subgroup differences (random effects) was not significant (*χ* ^2^_1_ = 0.31, *p* = 0.5801).

**Figure:**
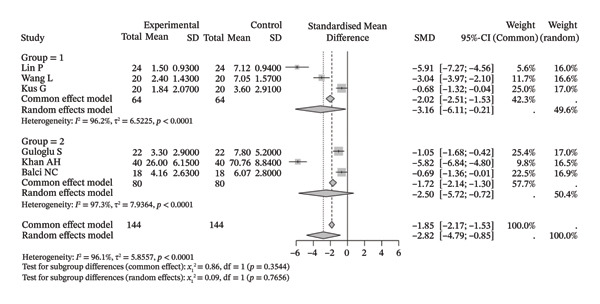
(a)

**Figure:**
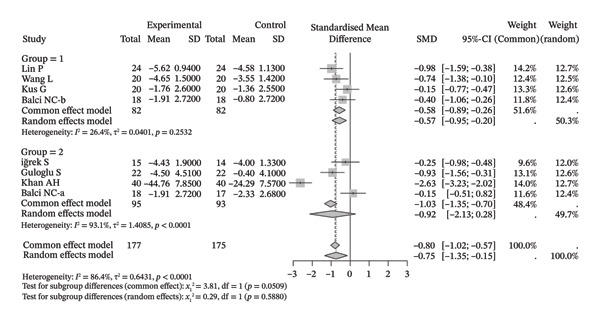
(b)

Seven studies (8 samples) were included in the meta‐analysis comparing shoulder pain between the two groups (159 in the intervention group vs. 175 in the control group). Compared with those of the control group, the values of improvement in the neuromuscular physiotherapy group were significantly greater (Figure 2(b)) than those of the control group after treatment (SMD [95% CI] = −0.75 [−1.35, −0.15], *I* ^2^ = 86.4%). Both the SMD [95% CI] = −0.58 [−0.89, −0.26], *I* ^2^ = 26.4%) and the other rehabilitation methods (SMD [95% CI] = −0.92 [−2.13, 0.28], *I* ^2^ = 93.1%) tended to reduce shoulder pain in the neuromuscular physiotherapy group compared with the control group.

### 3.3. Shoulder Range of Motion

Several meta‐analyses on improvements in shoulder range of motion included three to six studies each. The specific results are shown in Table 2. Overall, neuromuscular physical therapy techniques can improve the range of motion of the shoulder joint in terms of flexion (Figure 3(a)), extension (Figure 3(b)), abduction (Figure 3(c)), external rotation (Figure 3(d)), and internal rotation (Figure 3(e)). Subgroup analysis also suggested that, regardless of whether this technique was used alone or in combination with other rehabilitation techniques, there was a tendency to improve the range of motion of the shoulder joint.

**TABLE 2:** Results of range of motion.

	**Improvement value of the intervention group**			**The improvement value compared with the control group**		
	**Included studies**	**I** ^2^ **(%)**	**Main analysis results**	**Included studies**	**I** ^2^ **(%)**	**Main analysis results**
Forward flexion	5	80.10	1.50 [0.75, 2.24]	6	68.00	0.44 [−0.04, 0.91]
Extension	3	85.50	1.14 [0.12, 2.16]	4	13.30	0.32 [−0.01, 0.65]
Abduction	5	91.30	2.05 [0.66, 3.43]	6	83.90	0.19 [−0.51, 0.90]
External rotation	4	88.90	2.00 [0.46, 3.54]	5	68.30	0.68 [0.11, 1.25]
Internal rotation	3	90.60	1.76 [0.29, 3.22]	4	76.10	0.24 [−0.46, 0.94]

*Note:* A positive SMD indicates a greater degree of improvement in the intervention group.

FIGURE 3Forest plots of improvement in shoulder range of motion after treatment. Meta‐analysis shows that neuromuscular physiotherapy significantly improved (a) flexion, (b) extension, (c) abduction, (d) external rotation, and (e) internal rotation compared to baseline. The pooled random‐effects SMDs with 95% CIs were as follows: (a) SMD = 1.50 (95% CI: 0.75, 2.24), *I* ^2^ = 80.1%, *τ* ^2^ = 0.5910, *p* = 0.0005; (b) SMD = 1.14 (95% CI: 0.12, 2.16), *I* ^2^ = 88.5%, *τ* ^2^ = 0.6930, *p* = 0.0010; (c) SMD = 2.05 (95% CI: 0.66, 3.43), *I* ^2^ = 91.3%, *τ* ^2^ = 2.3132, *p* < 0.0001; (d) SMD = 2.00 (95% CI: 0.46, 3.54), *I* ^2^ = 88.9%, *τ* ^2^ = 2.2497, *p* < 0.0001; (e) SMD = 1.76 (95% CI: 0.29, 3.22), *I* ^2^ = 90.6%, *τ* ^2^ = 1.5181, *p* < 0.0001.

**Figure:**
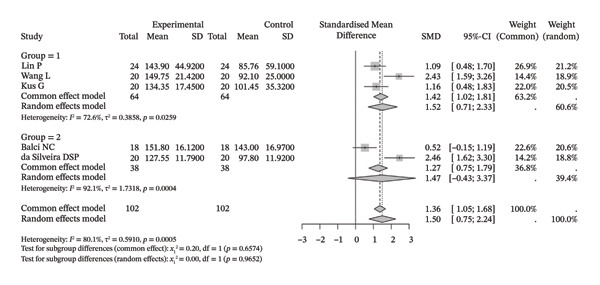
(a)

**Figure:**
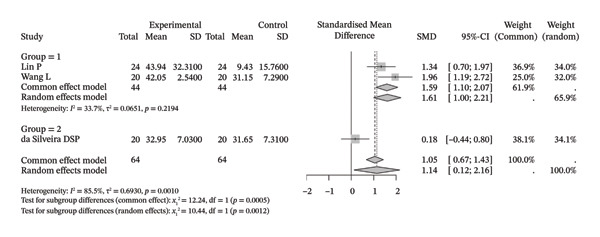
(b)

**Figure:**
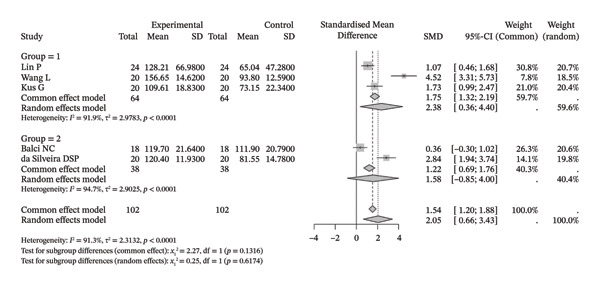
(c)

**Figure:**
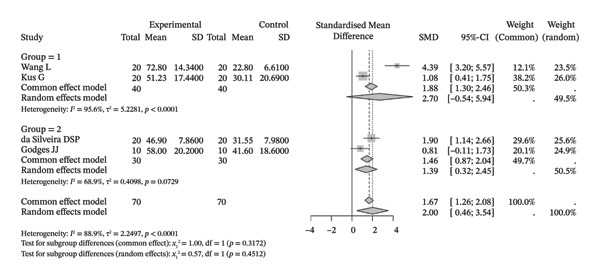
(d)

**Figure:**
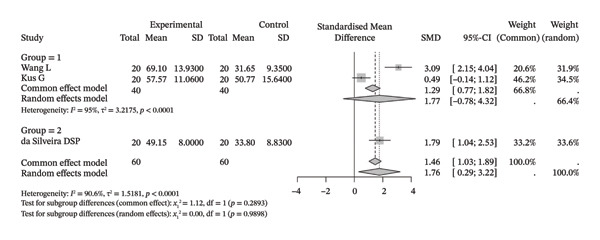
(e)

Compared with the control group, neuromuscular rehabilitation techniques were more effective in improving the external rotation range of motion of the shoulder joint (Figure 4(d)) and tended to improve the flexion and extension ranges of motion of the shoulder joint (Figures 4(a), 4(b)), whereas there was no significant difference in improving abduction (Figure 4(c)) or internal rotation (Figure 4(e)) compared with the control group.

FIGURE 4Forest plots comparing the improvement in shoulder range of motion between neuromuscular physiotherapy and control groups for (a) flexion, (b) extension, (c) abduction, (d) external rotation, and (e) internal rotation. The overall random‐effects meta‐analysis showed significant improvements for (a) flexion (SMD = 0.44, 95% CI: −0.04 to 0.91; *I* ^2^ = 68.0%, *τ* ^2^ = 0.2904, *p* = 0.0046), (b) extension (SMD = 0.31, 95% CI: −0.04 to 0.67; *I* ^2^ = 13.3%, *τ* ^2^ = 0.0212, *p* = 0.3260), (c) abduction (SMD = 0.19, 95% CI: −0.51 to 0.90; *I* ^2^ = 83.9%, *τ* ^2^ = 0.7733, *p* < 0.0001), (d) improvements for external rotation (SMD = 0.68, 95% CI: 0.11 to 1.25; *I* ^2^ = 68.3%, *τ* ^2^ = 0.2749, *p* < 0.0134), and (e) internal rotation (SMD = 0.24, 95% CI: −0.46 to 0.94; *I* ^2^ = 76.1%, *τ* ^2^ = 0.3824, *p* = 0.0057) were not statistically significant.

**Figure:**
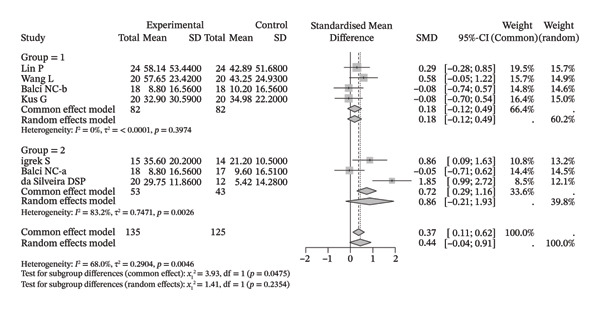
(a)

**Figure:**
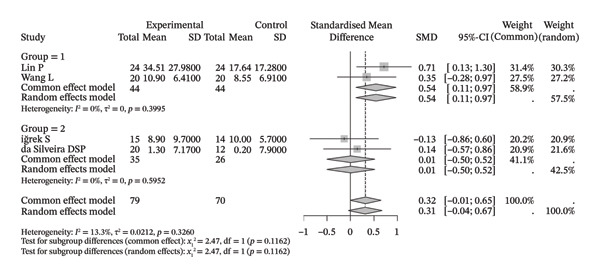
(b)

**Figure:**
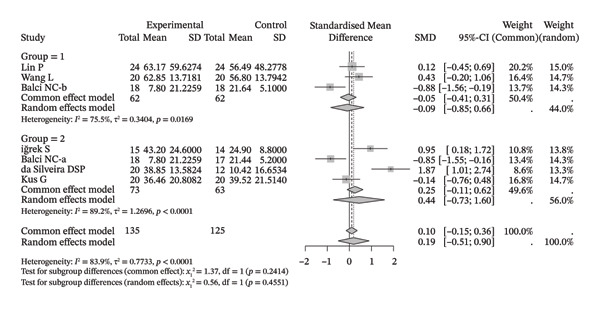
(c)

**Figure:**
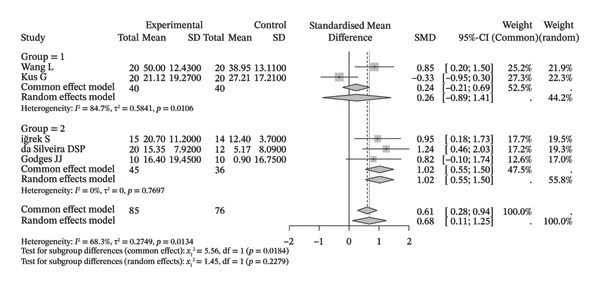
(d)

**Figure:**
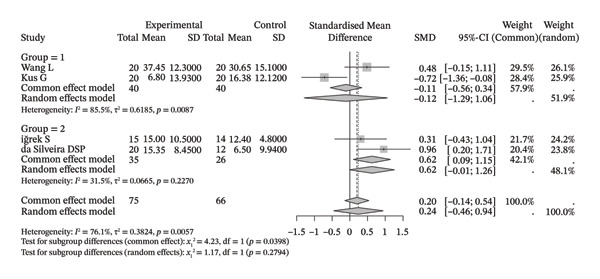
(e)

### 3.4. Function/Disability

A total of six original studies (130 participants) were included in the meta‐analysis on function/disability (Figure 5(a)). The results indicated that neuromuscular physical rehabilitation techniques could improve the related functions/disabilities of shoulder disorders (SMD [95% CI] = −1.95 [−3.71, −0.19], *I* ^2^ = 94.4%). Moreover, subgroup analysis suggested that, regardless of whether this technique was used alone (SMD [95% CI] = −1.18 [−1.70, −0.65], *I* ^2^ = 30.9%) or in combination with other rehabilitation techniques (SMD [95% CI] = −2.37 [−5.07, 0.33], *I* ^2^ = 96.5%), there was a tendency to improve the functions/disabilities of patients with shoulder disorders.

FIGURE 5Results of function/disability. (a) Symptom improvement after treatment. The overall pooled random‐effects SMD was −1.95 (95% CI: −3.71 to −0.19), indicating a significant reduction in pain with high heterogeneity (*I* ^2^ = 94.4%, *τ* ^2^ = 4.6973, *p* < 0.0001). Subgroup analysis showed significant improvements both when the intervention was used alone (Group 1: SMD = −1.17, 95% CI: −1.80 to −0.54; *I* ^2^ = 30.9%, *τ* ^2^ = 0.0639, *p* = 0.2291) and in combination with other methods (Group 2: SMD = −2.37, 95% CI: −5.07 to 0.33; *I* ^2^ = 96.5%, *τ* ^2^ = 7.3966, *p* < 0.0001), with no significant subgroup differences (random effects: *χ* ^2^_1_ = 0.72, *p* = 0.3951). (b) Comparison with the control group after treatment. The overall pooled random‐effects SMD was −0.65 (95% CI: −1.25 to −0.05), demonstrating superior pain relief compared to control with substantial heterogeneity (*I* ^2^ = 87.1%, *τ* ^2^ = 0.7124, *p* < 0.0001). Subgroup analysis confirmed benefits for the intervention alone (Group 1: SMD = −0.31, 95% CI: −0.74 to 0.11; *I* ^2^ = 32.8%, *τ* ^2^ = 0.0604, *p* = 0.2155) and suggested a greater, albeit nonsignificant effect for the combined approach (Group 2: SMD = −0.88, 95% CI: −1.89 to 0.13; *I* ^2^ = 91.8%, *τ* ^2^ = 1.2055, *p* < 0.0001), with no significant subgroup differences (random effects: *χ* ^2^_1_ = 1.03, *p* = 0.3092).

**Figure:**
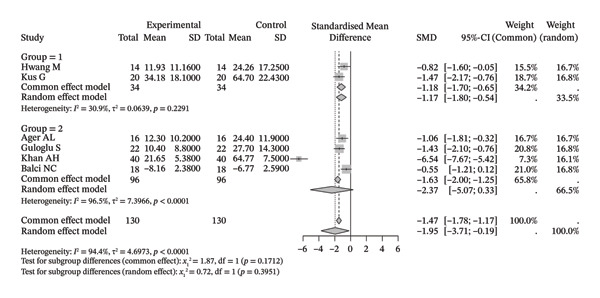
(a)

**Figure:**
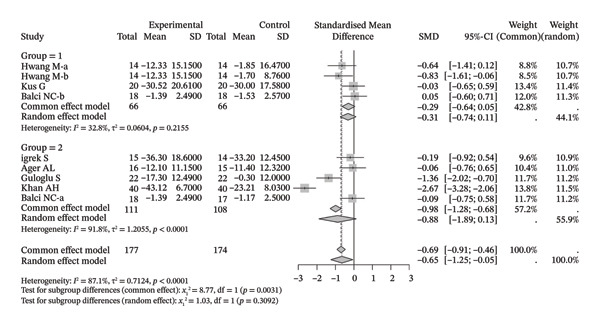
(b)

Seven studies (9 samples) were included in a meta‐analysis comparing improvements in shoulder dysfunction/disability with those in the control group (Figure 5(b)). Compared with the control group, neuromuscular physical rehabilitation techniques were superior in improving shoulder dysfunction/disability (SMD [95% CI] = −0.65 [−1.25, −0.05], *I* ^2^ = 87.1%). Moreover, subgroup analysis suggested that, whether used alone (SMD [95% CI] = −0.29 [−0.64, 0.05], *I* ^2^ = 32.8%) or in combination with other rehabilitation techniques (SMD [95% CI] = −0.88 [−1.89, 0.13], *I* ^2^ = 91.8%), there was a trend toward superiority over the control group.

### 3.5. Bias Assessment

**FIGURE 6:**
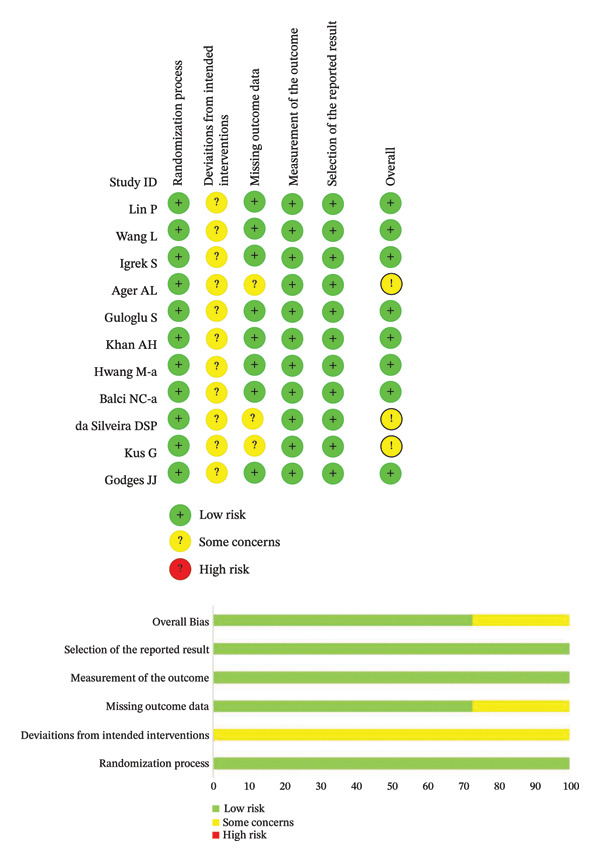
Evaluation of literature quality.

Bias in the original studies: The RoB2 method was used to evaluate 5 bias domains (Figure 6). Overall, the degree of selection bias in all original studies was low, and the risk of intervention deviation in all original studies was unclear. Additionally, there was a risk of missing outcome data in 3 original studies by Ager et al. [21], da Silverira et al. [15], and Kus et al. [19], because they did not report the preintervention outcome data. The measurement bias in all original studies was low, and the reporting bias in all original studies was low. Overall, the overall risk of bias of the 11 original studies was low.

Publication bias: Funnel plot analysis revealed publication bias (Figure 7). Most of the study points were distributed on both sides of the central axis of the funnel plot, showing no obvious asymmetry trend. However, there were some results where a few study points deviated from the center, which might be related to heterogeneity in sample size and small sample effects. Overall, no significant publication bias was found, but small sample studies may have had an impact on the overall results due to unstable effect size estimation.

FIGURE 7Publication bias. (a) Pain. (b) Flexion. (c) Extension. (d) Abduction. (e) External rotation. (f) Internal rotation. (g) Function/disability. Footnote 1 refers to after treatment. Footnote 2 indicates comparison with the control group.

**Figure:**
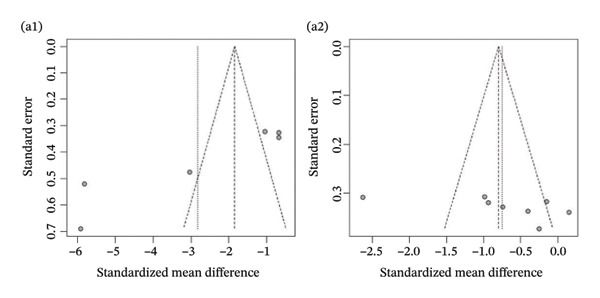
(a)

**Figure:**
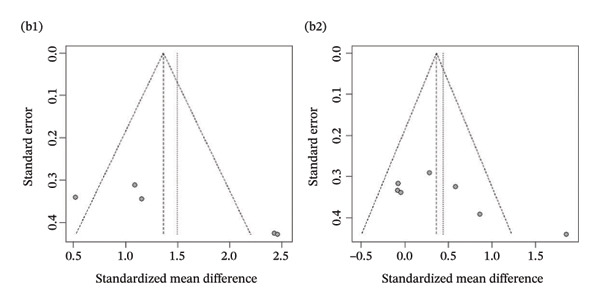
(b)

**Figure:**
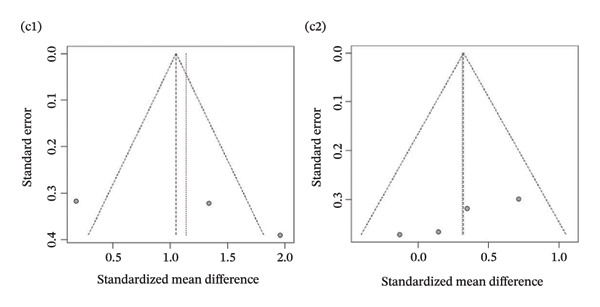
(c)

**Figure:**
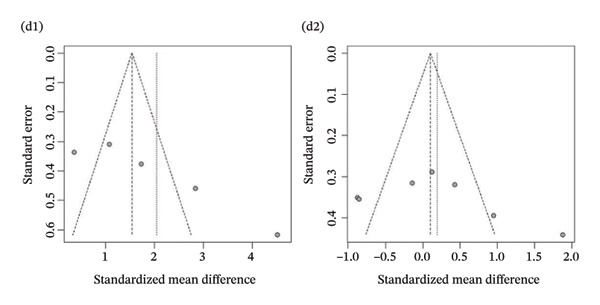
(d)

**Figure:**
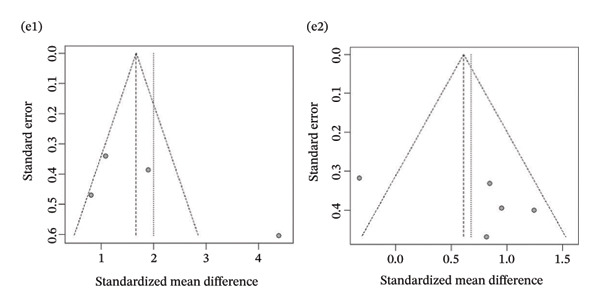
(e)

**Figure:**
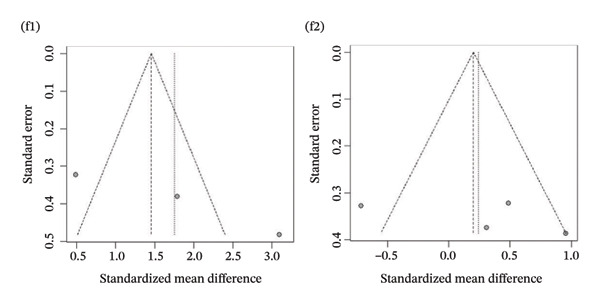
(f)

**Figure:**
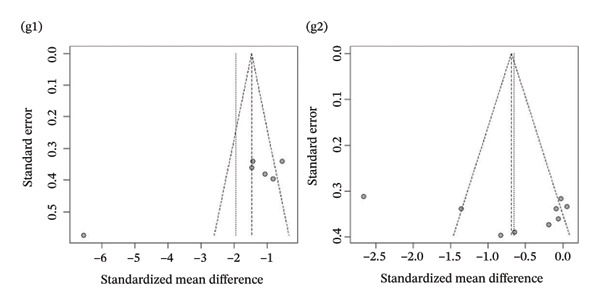
(g)

Overall, although there was high heterogeneity among the studies, the results of the sensitivity analysis were consistent with those of the main analysis.

## 4. Discussion

This study conducted a systematic review and meta‐analysis of 11 RCTs to investigate the effects of neuromuscular physical therapy (mainly PNF techniques) on pain, range of motion, and function in shoulder‐related disorders. The results revealed that neuromuscular physical therapy significantly improved shoulder pain, and the degree of improvement in pain was greater in the experimental group than in the control group. In terms of shoulder joint range of motion, this therapy had positive effects on forward flexion, backward extension, abduction, external rotation, and internal rotation of the shoulder. The improvement in the external rotation range of motion was more significant than that in the control group. In terms of function/disability, neuromuscular physical therapy improved the function/disability status of the shoulder and had an advantage over the control group. These findings provide evidence supporting the application of neuromuscular physical therapy in the rehabilitation of shoulder disorders.

Neuromuscular rehabilitation is a training method aimed at improving muscle function and enhancing athletic performance. Some previous studies have shown that neuromuscular interventions after ankle and knee joint injuries can effectively prevent recurrent injuries and improve joint function [25]. Moreover, neuromuscular facilitation techniques can help patients with scapulohumeral periarthritis relieve pain, improve shoulder function, and enhance their ability to perform activities of daily living [26].

In the field of shoulder rehabilitation, pain, limited range of motion, and functional impairment are the main problems faced by patients and seriously affect their quality of life and daily activities [6]. This study confirmed that neuromuscular rehabilitation techniques can significantly improve shoulder pain and relieve patients who have been suffering from pain for a long period of time. This is crucial for improving patients’ comfort, reducing activity avoidance behaviors caused by pain, and is essential for enhancing patients’ ability to perform complex tasks, such as dressing, combing hair, raising hands, and reducing the impact of limited activities on their lives [7]. For patients with periarthritis of the shoulder, improved joint range of motion means that they can move their shoulders more freely, reducing the inconvenience of daily life. From the perspective of functional recovery, neuromuscular rehabilitation techniques can effectively improve shoulder function/disability and enhance patients’ ability to perform complex tasks, such as light housework and simple work, helping patients better return to family and society [7, 11]. In clinical practice, doctors and rehabilitation therapists can incorporate neuromuscular rehabilitation techniques into treatment plans for patients with shoulder disorders on the basis of the results of this study, providing patients with more effective rehabilitation options, promoting the recovery of shoulder function, improving quality of life, and reducing the risk of long‐term disability caused by shoulder disorders.

Our study extends the existing body of evidence through a comprehensive approach. In contrast to previous research, this meta‐analysis synthesizes evidence across a spectrum of shoulder disorders, including adhesive capsulitis, subacromial impingement syndrome, rotator cuff tendinopathy, proximal humerus fractures, and postmastectomy lymphedema. This integrative design is a key point of distinction, as it enables us to evaluate the broader applicability of neuromuscular physiotherapy, primarily PNF techniques, in shoulder rehabilitation.

Despite differences in scope, our core findings are consistent with and reinforce the conclusions of prior focused reviews. For instance, the significant improvements in pain and function we observed align with the results reported by Zhu et al. [26] regarding scapulohumeral periarthritis. This consistency across different study designs enhances the credibility of the conclusion that neuromuscular rehabilitation is beneficial for shoulder disorders. A potential advantage of our comprehensive analysis is that it suggests the therapeutic effects of PNF may transcend specific diagnoses, possibly by addressing common underlying impairments in neuromuscular control and proprioception shared by various shoulder pathologies. This provides a theoretical rationale for its wider clinical application, particularly where differential diagnosis can be complex.

However, the comprehensive nature of our analysis also means that the pooled effect for any single disorder category is derived from a relatively small number of studies. Future research should incorporate larger‐scale RCTs for each specific disorder to obtain more precise effect estimates. Nonetheless, our study offers valuable preliminary evidence supporting the use of neuromuscular physiotherapy as an effective adjunctive therapy across a range of common shoulder‐related conditions.

However, it is also necessary to be aware of the limitations of this study. First, the sample sizes of the original studies included in this research were relatively small, and even after the meta‐analysis and the combined sample size were calculated, the sample size was still not large. Larger‐scale clinical trials are necessary in the future. Second, owing to the differences in the reported results in the original studies, this research did not include patients’ psychological and quality‐of‐life aspects as one of the outcomes. However, these two characteristics should be taken into account in clinical practice. Finally, this study lacked the assessment of the long‐term efficacy of neuromuscular rehabilitation techniques. In the future, the follow‐up time should be increased to explore its long‐term effects.

In conclusion, this study revealed that neuromuscular physical therapy can effectively relieve pain in patients with shoulder disorders, improve joint mobility, and enhance function. These findings support its use as an adjunctive therapy in conventional rehabilitation, but its limitations lie in the small sample size in the original studies and the lack of long‐term follow‐up. Future research should explore larger sample groups, patient self‐reported quality of life, and long‐term efficacy.

## 5. Conclusions

This systematic review and meta‐analysis highlights the effectiveness of neuromuscular physical therapy, particularly PNF, in the rehabilitation of shoulder‐related disorders. The findings show significant pain relief, improved shoulder range of motion, especially in external rotation, and enhanced shoulder function, with superior outcomes compared to control treatments. These results support the use of neuromuscular rehabilitation as an adjunct to conventional therapies for shoulder disorders. However, due to small sample sizes and a lack of long‐term follow‐up, further large‐scale studies are needed to confirm the long‐term benefits of these techniques. This study provides valuable evidence for improving clinical rehabilitation practices and patients’ quality of life.

## Author Contributions

Conceptualization, Yingkai Zhang; writing–original draft preparation, Dan Lu and Yan Lan.

## Funding

This work was supported by the Zhejiang Province Traditional Chinese Medicine Science and Technology Plan Project (Project# 2025ZX370).

## Disclosure

All authors have read and agreed to the published version of the manuscript.

## Ethics Statement

The authors have nothing to report.

## Consent

The authors have nothing to report.

## Conflicts of Interest

The authors declare no conflicts of interest.

## Data Availability

The data that support the findings of this study are available from the corresponding author upon reasonable request.
